# Study protocol: an effectiveness, cost-effectiveness, and process evaluation of *headspace* Denmark

**DOI:** 10.3389/fpubh.2025.1491756

**Published:** 2025-04-07

**Authors:** Siv T. B. Bjørkedal, Thomas N. Christensen, Rie M. Poulsen, Anne Ranning, Anne A. E. Thorup, Merete Nordentoft, Anders Bo Bojesen, Lene H. Hastrup, Marte Ustrup, Lene F. Eplov

**Affiliations:** ^1^Copenhagen Research Center for Mental Health (CORE), Hellerup, Denmark; ^2^Copenhagen Research Unit for Recovery, Copenhagen, Denmark; ^3^National Board of Social Services in Denmark, Odense, Denmark; ^4^Department of Psychology, University of Copenhagen, Copenhagen, Denmark; ^5^Research Unit, Child and Adolescent Mental Health Center, Hellerup, Denmark; ^6^Department of Clinical Medicine, Faculty of Health Sciences, University of Copenhagen, Copenhagen, Denmark; ^7^Psychiatric Research Unit, Psychiatry in Region Zealand, Slagelse, Denmark; ^8^Danish Centre for Health Economics (DaCHE), University of Southern Denmark, Odense, Denmark

**Keywords:** youth mental health, volunteerism, complex intervention, civic society, anti-stigma

## Abstract

**Introduction:**

Since 2013, *headspace* Denmark has been offered in specific areas to adolescents and young adults between 12 and 25 years, to promote youth mental health and wellbeing. *Headspace* provides free counselling and support, primarily delivered by trained volunteers in the *headspace* centres and provides information and knowledge about youth mental health, and *headspace* services, through community engagement. Until now, effectiveness evaluation of the Danish *headspace* centres has not been conducted.

**Methods:**

Present study consists of (1) an effectiveness evaluation designed as a propensity score matched quasi-experimental trial, where the exposed person (*n* = 1,500), in this case the young person receiving counselling sessions at *headspace*, will be matched by using propensity scores to six unexposed individuals. (2) A cost-effectiveness evaluation (3) a process evaluation with predominantly qualitative methods to investigate the implementation of key activities of *headspace,* their mechanisms of change, and interactions with contextual factors.

**Discussion:**

*headspace* centres have achieved national endorsement and are implemented in 30 municipalities in Denmark. Thus, there is a need to investigate the effectiveness of the services. Results from the evaluation can also contribute to new knowledge targeted at international youth mental health promotion initiatives. However, this evaluation is limited by selection bias since it is not possible to separate the impact of the intervention from the impact of help-seeking behaviour.

## Introduction

Increasing rates of poor mental health among adolescents and young adults is a serious, global public health concern (1, 2). About 62.5% of all mental illnesses have their onset before the age of 25 years (3) and are considered the leading cause of disability in most European countries (4). In Denmark, youth mental health is a pivotal public health challenge (5). According to the Danish Health Authorities, more than 73,000 children and adolescents (corresponding to 63 out of 1,000 youths) are living with a mental illness, which is a 39% increase over the last 10 years (6). Furthermore, national surveys on health and wellbeing in persons up to 24 years of age show that although most young people in Denmark perceive their health to be good, there is an increasing proportion of adolescents and young adults reporting mental health complaints such as high levels of stress and emotional problems (5). There is no doubt that mental health problems pose a threat to young people’s health, wellbeing, and opportunities for thriving and living fulfilling lives. Mental health problems in childhood and adolescent years are also associated with health deterioration, e.g., recurrent illness episodes and functional impairment (5), and obstruction of educational and vocational attainment (7, 8). Thus, poor mental health in youth does not only have adverse consequences for the individual but also for society in terms of loss of productivity and increased expenses to health and social services (1, 9–11).

In Denmark, the health care system is publicly funded, which means that mental health services are available to everyone, free of charge. Despite this, a gap exists between the growing group of young people with mental health complaints and those who receive sufficient support in the mental health system. Barriers for young people to access traditional mental health services include stigma towards mental health problems, long waiting lists for assessment and treatment, and lack of mental health literacy (12, 13). Investments in preventive and mental health promotion initiatives are necessary to help and support young people with mental health complaints before more severe mental health problems develop with the risk of consequently social and/or vocational marginalisation.

To address the above-mentioned challenges and to empower young people with information about mental health, and opportunities for support, the Non-Governmental Organisation, Det Sociale Netværk, has during the previous decade established 30 *headspace* centres in municipalities in all Danish Regions. The *headspace* centres provide a low threshold and easily accessible supportive place regarding mental health, to promote mental health and wellbeing in youth between 12 and 25 years. The *headspace* centres have a “soft entry” to make it as easy as possible for young people to get help to any problem they have, that may affect their mental health and wellbeing. In Denmark, the *headspace* centres are based on and branded like the Australian *headspace* model, but contextual adaptations have been made, to meet local needs (14). The *headspace* centres offer free counselling services (“*someone to talk to*”) predominantly delivered by trained adult volunteers. The volunteers work in pairs, either with another volunteer, or an employed counsellor in the *headspace* centre. In contrast to the Australian *headspace* model, mental health assessment and treatment are not provided. If the counsellor finds that the person may need, for instance, clinical treatment, the person is supported to access these services. This is referred to as bridge building in *headspace*.

The dissemination of *headspace* centres in Denmark necessitates evidence-based knowledge on whether *headspace* is an effective intervention to promote mental health and wellbeing among young people. It also necessitates knowledge on the costs and outcomes of *headspace*. An evaluation of the impact of *headspace* in Denmark on young people’s lives and a cost-consequence analysis was conducted by a consulting agency in 2019 (15). Findings showed that 94% of the youth receiving counselling in *headspace* reported feeling understood and respected. Additionally, the evaluation indicated that counselling sessions improved the wellbeing and life satisfaction of the participants. According to the Cantril’s ladder (16), there was an average of 9% improvement in life satisfaction, while the General Population - Clinical Outcomes in Routine Evaluation (CORE-GP) (17) scale showed an average 7% rise in mental wellbeing. Additionally, the proportion of people who reported feeling lonely reduced by 9% according to the Three-Item Loneliness Scale (18). However, it was emphasised that a major limitation was the lack of a comparable control group, which meant that no firm conclusions could be drawn regarding the effects of *headspace.* The analysis of cost and returns to society was based on *headspace* operating costs and scenario-based analyses where the derivative effects for the young people were estimated. Based on the evaluation, it was therefore not possible to draw any conclusion on the cost-effectiveness of *headspace* due to the short follow-up period, the lack of a control group, and the lack of register-based health care and municipal social care costs (15).

A 2022 national evaluation of headspace in Australia utilised a pre-post treatment comparison design, with regression to mean adjustments, by using the variation in outcomes measured at intake and before the second session. The results showed improvement across all outcomes: wellbeing, functioning and quality of life. In 52% of the young people the improvement was clinically significant in at least one of the outcomes (19). Findings indicated that higher session attendance was associated with greater reductions in distress, as measured by the Kessler Psychological Distress Scale (K10), and improvements in psychosocial functioning, assessed through the Social and Occupational Functioning Assessment Scale (SOFA). For instance, young people who attended two sessions showed minimal change (−0.1 K10; +0.5 SOFA), whereas those attending 3–5 or 6–9 sessions experienced greater improvements (−1.5 and −2.2 K10; +2.7 and +5 SOFA, respectively). The Australian evaluation cannot be directly compared to the Danish evaluation conducted by Rambøll, as the studies measured different outcomes using distinct methodologies. However, the two evaluations suggest that improvements are attainable, in outcomes related to youth mental health. Matthay et al. (20) emphasise that effect sizes vary based on intervention features (intensity, duration, content, and implementers) and mechanisms. Unlike Australia’s primary care-based model, staffed by health professionals and offering treatments such as cognitive behavioural therapy and medical consultations, *headspace* centres in Denmark operate as a volunteer-driven civic initiative without mental health specialists, making it a lower-intensity, non-medical intervention. Thus, smaller effect sizes than the ones identified in the Australian evaluation may be expected in this evaluation of *headspace* in Denmark. However, small effect sizes can also be important, from a mental health promotion and prevention perspective (20). Thus, more scientifically based knowledge is needed about if and how the *headspace* intervention can enhance youth mental health and wellbeing and prevent mental illness. There is also a need for documentation of the cost-effectiveness of the intervention where all relevant costs are measured. This is important to inform policymakers in deciding future investments and implementation of preventive mental health interventions like *headspace* to ensure that resources are being used properly. Moreover, there is a growing need to investigate the key activities of the Danish *headspace* intervention and to document how these can be implemented in a local context and how they might create the expected effects on youth mental health and wellbeing.

The overall aim of present evaluation is to investigate the potential effects on young individuals in the presence of *headspace* entities offering easy access and counselling for help-seeking youth, as well as the cost-effectiveness of the intervention. In addition, the aim is to investigate key activities of the *headspace* intervention utilising a process evaluation and analyse how the key activities can support the intended effects in different local contexts.

### *headspace* Denmark

**Figure 1:**
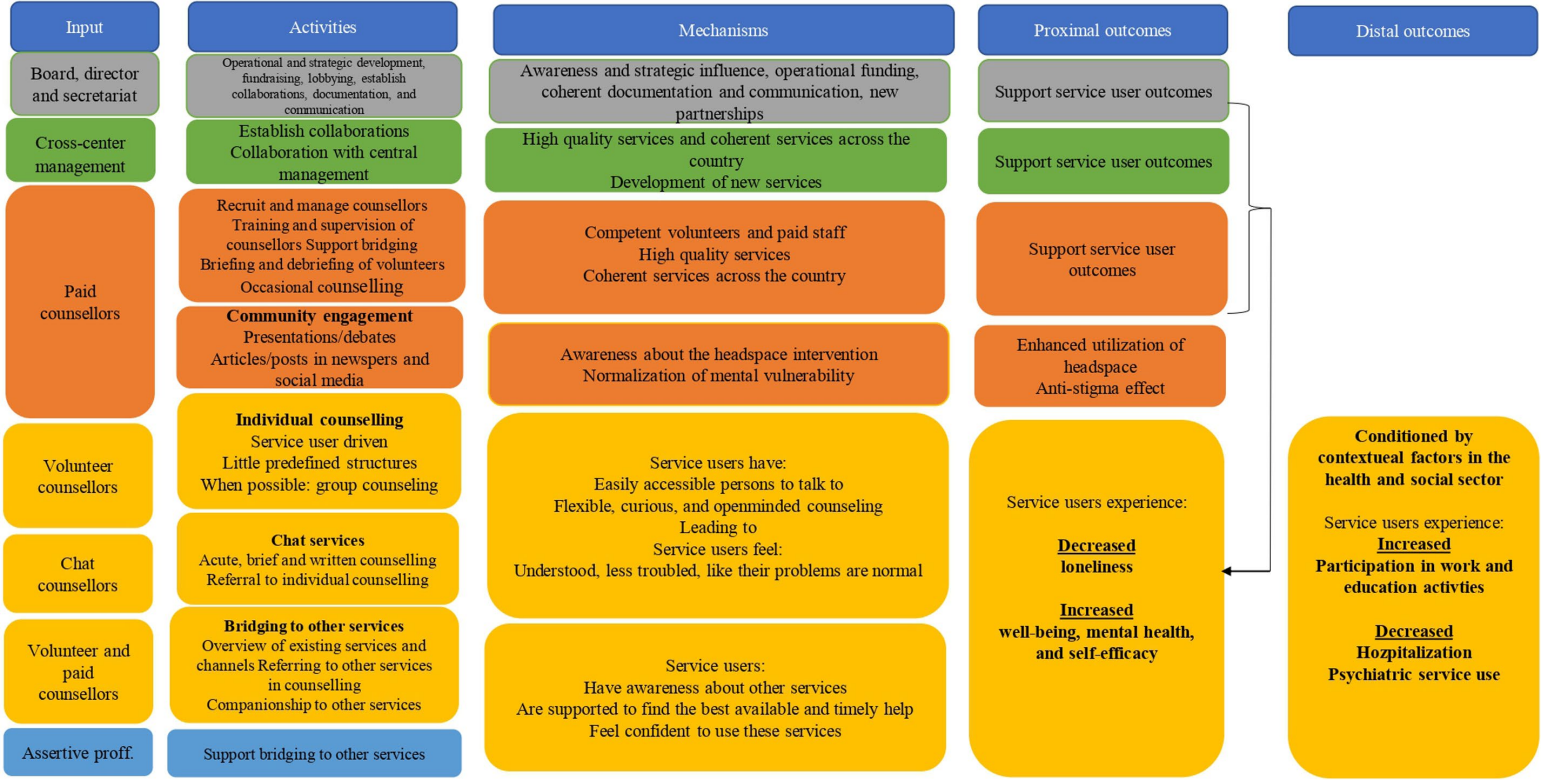
Logic model of the headspace intervention. The figure provides an overview of (1) the activities in headspace; yellow activities are in direct contact with service users, whereas grey, green, orange, and blue activities primarily support the yellow activities, (2) the inputs (personnel) that are necessary to deliver the activities (3) the mechanisms by which the key activities are expected to create the intended outcomes, and (4) the expected proximal and distal outcomes. Yellow outcomes are directly related to service users, while the green and orange outcomes support the yellow proximal outcomes as indicated by the arrow.

The *headspace* intervention can be mapped as a complex intervention since it consists of several flexible components that interact to produce change, involves many types of stakeholders and organisations, and is expected to create multiple outcomes. To accommodate this complexity, the study is guided by the Medical Research Council (MRC) guidelines for evaluation of complex interventions (41). Consequently, the study takes a point of departure in a figurative programme theory for the *headspace* intervention (see Figure 1). The preliminary programme theory was developed for this study in collaboration with central *headspace* management and is based on internal documents in the Danish *headspace* initiative.

### *headspace* centres

*headspace* Denmark consists of 30 *headspace* centres and one national chat centre. All centres offer free counselling to young people between 12 and 25 years. The *headspace* centres are staffed with one cross-centre manager (one manager serves about three *headspace* centres), two paid counsellors, volunteer counsellors, and two part-time (15 h. per week) assertive professionals employed in the local mental health services and/or social services. The *headspace* centres are located so they are accessible to young people (for instance close to public transportation). The centres furniture and decorations signal a youth-friendly atmosphere. The *headspace* centres are open 3 days a week, between 12 and 18 (12 AM to 6 PM) and the chat centre is open between 12 and 22 (12 AM to 10 PM).

Training of counsellors involves an online training program, a weekend course, and on-site training and supervision with experienced counsellors and professionals. The counselling provided to the youth is delivered in an open-minded, curious, and flexible approach, and the counsellors are guided by a person-centred approach that sets the young person’s needs at the centre of their work. Volunteering is a key principle in the *headspace* organisation, and young people seeking counselling and support are informed that most counsellors are volunteers.

Young people accessing counselling do not need to state their name or personal ID number, so their identity remains anonymous. The *headspace* centres operate under the motto “Nothing is too big or too small,” meaning that all young people in the targeted age group can access the centres. Should the young person require additional support such as a mental health assessment, specialist treatment or support from social services, the counsellors can offer support and guidance in accessing these services. In addition to counselling and bridging to other services, *headspace* centres play an active role in providing information about youth mental health, de-tabooing mental health problems among young people, as well as making *headspace* centres visible and thus accessible through community engagement and collaborations with schools, etc.

## Overall study design

**Figure 2:**
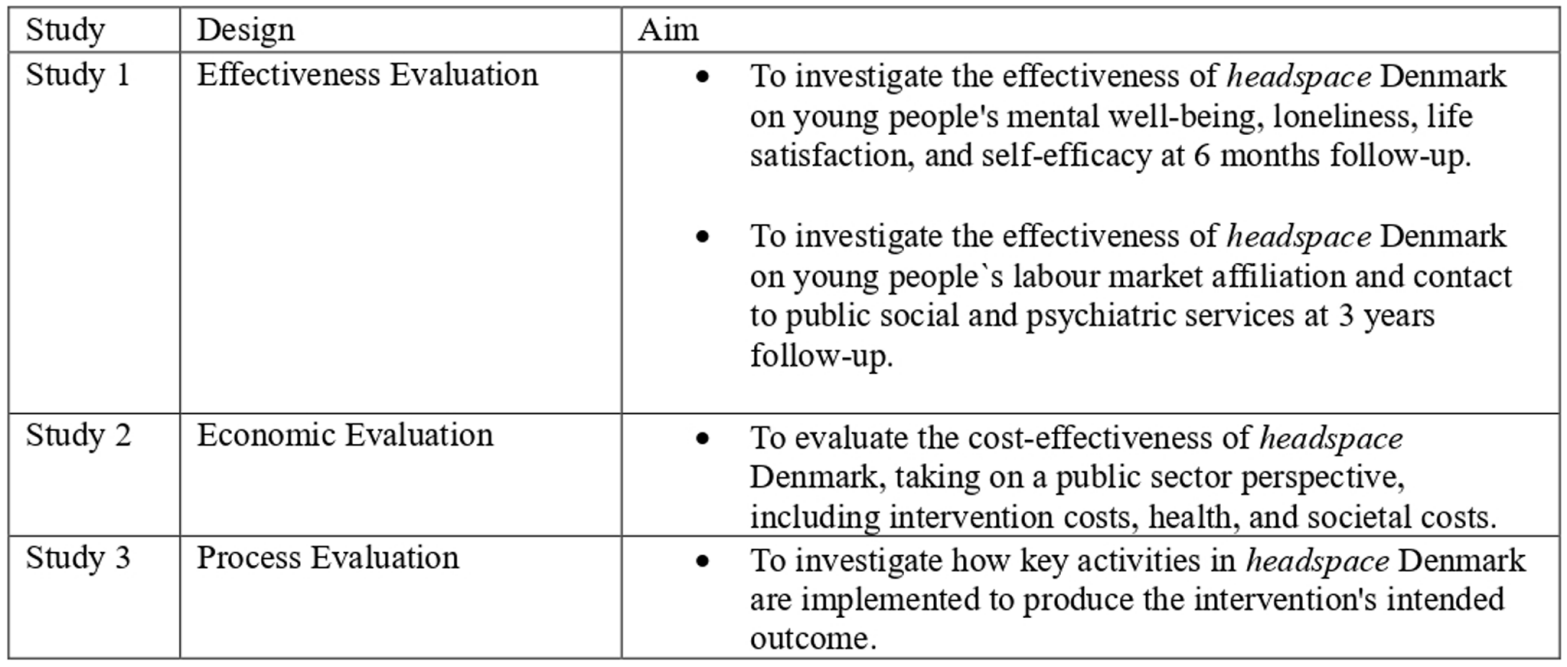
An overview of the three studies comprising the evaluation of headspace Denmark.

The study protocol consists of three studies: an effectiveness evaluation, a cost-effectiveness evaluation, and a process evaluation, to provide a holistic and comprehensive understanding of *headspace* Denmark, to determine whether the intervention meets it objectives, to investigate the costs of the intervention to the outcomes achieved, and to analyse how the intervention is implemented to improve youth mental health. An overview of the three studies is provided in Figure 2. Appendix 1 shows a timeline of the studies.

## Study 1: effectiveness evaluation

### Methods

#### Study design

The evaluation is designed as a propensity score matched quasi-experimental trial, meaning that a young person receiving counselling sessions at *headspace* will be matched by using propensity scores to six unexposed individuals (controls) based on several background characteristics with potential associations to *headspace* participation. The study will, by establishing a comparable control group, evaluate the results from the *headspace* group and measure whether *headspace* has any effect on mental wellbeing, loneliness, life satisfaction, and self-efficacy at 6 months follow-up, and measure the effects on labour market affiliation, and contact to public social and psychiatric services at 3 years follow-up.

#### Study sample

In total, 1,500 *headspace* service users are aimed for being included during the first 15 months of the study. The inclusion criteria for the intervention group are age between 12 and 25, participation in at least one counselling session in one of the physical national *headspace* centres, and willingness to give informed consent to participate in the study. Since some young people will have had one or more sessions at a *headspace* centre before the data collection begins, it is required that the first recorded measurement of young people is also their first actual session at a *headspace* centre. Young people attending the first session in *headspace* receive oral and written information about the study, from the counsellors. They are informed about the study’s purpose and procedures, including using questionnaires and register data. It is stressed that participation in the study is voluntary, and that declining to participate does not have any negative consequences for them, e.g., their right to receive counselling in *headspace.* Persons wanting to participate in the study are given a link, where they can log in to provide personal information (e.g., unique personal ID number, contact information), give informed consent and fill out questionnaires. Youth below 15 years are provided with a link to give to their parents/caregivers who need to provide consent before the person can participate in the study.

All included participants will be registered with their unique personal ID number which allows for individual-level linkage of information across nationwide registers provided by Statistics Denmark. Young people who do not want to provide their unique personal ID number cannot participate in the study. As *headspace* collects basic information on users, a supplementary analysis will be made comparing *headspace* users accepting registration on unique personal ID with *headspace* users not accepting to give their personal ID and participate in this evaluation. This comparison includes age, gender, and additional information on social and health-related problems faced by participants.

#### Procedures for matching the control group

The matching procedure must ensure that the control group is selected on significant prognostic characteristics, related to the young person contacting the *headspace* centre, which are like a participant in *headspace*. After 6 and 15 months of the 15 months inclusion period, controls are matched to the included *headspace* users using propensity score matching. Approximately 10,000 controls in total, living outside of the current *headspace* catchment area, will be matched. The repeated matching procedure is to ensure that the time lag between the *headspace* users’ survey response and the matched control can be reduced to 6 months. Table 1 presents an overview of the planned factors and registers in the matching.

**Table 1:** Variables planned to be included in the matching procedure.

Measure	Register name and provider
Psychiatric Outpatient courses Psychiatric Outpatient visits Psychiatric Hospitalisation	National Patient Register (LPR, LPR3, LPR-PSYK), The Danish Health Data Authority
Diagnosed psychiatric illness	National Patient Register (LPR), The Danish Health Data Authority
Preventive efforts to children provided by social services, which are initiated to avoid placement out of home	Statistics Denmark, Preventive efforts to children provided by social services (BUFO, BUU)
Placement out of home	Statistics Denmark, Children and youth placed out of home (BUAF)
Wellbeing in primary, short-term tertiary, and upper secondary school	Wellbeing surveys in primary, short-term tertiarty and upper seconday school. National Agency for It and Learning
Absenteeism from school	Absenteeism from school National Agency for It and Learning
National school tests, (math and Danish)	National school tests, National Agency for It and Learning Final grades for short-term tertiary and upper secondary school. (UDG), Statistics Denmark,
Parental income	Statistics Denmark (IND)
Parental employment and income transfer	Statistics Denmark (DREAM, AKM)
Parental marital status	Statistics Denmark (CIV)
Household type Parental ethnic background Sibling counts Parental civil status (diseased, divorced, etc.) Address change counts	Statistics Denmark (BEF, FTBARN, FTFORAEL)
Morbidity and health care use Parental morbidity and health care use	National Patient Register (LPR, LPR3), The Danish Health Data Authority The National Health Insurance Service Registry (SSR)
Primary health care use and chronic illness management	The National Health Insurance Service Registry (SSR)
Educational activities	Statistics Denmark (UDDA)
Delinquency and incarceration Parental delinquency and incarceration	The Danish Central Crime Register (KRAF) The Danish Central Crime Register (KRMS)
Prescription drug use	The Register of Pharmaceutical Sales (LSR)
Parental psychiatric illness, and substance use	National Patient Register (LPR) The Danish Health Data Authority
Disability services received	Handicap (HANDIC)
Neighbourhood effects, aggregated measures of: Unemployment Income Family types	Derived from above sources

Propensity scores are estimated using regularised logistic regression (11), or a low-depth random forest with *headspace* participation as a binary outcome. The matching will be done using the probability score and an optimal matching algorithm that will select the six closest matches with a caliper threshold at or below 0.25 on the log (odds) transformation of the propensity score. No replacements will be used. Missing values in predictors are handled by adding missingness indicator categories to categorical data and by replacing missing data with model-based imputations or slicing numerical variables in quantiles and then adding a categorical indicator of missingness. Matching will be carried out in two repeated batches with no overlap or cross-sample replacement. When all baseline data is collected, the matching is reiterated for the whole pool of cases and controls to ensure optimal balance on all matching parameters, at the cost of a smaller control group. The matching parameters will include all self-reported outcomes (e.g., wellbeing, loneliness) and register-based outcomes (e.g., number of hospitalizations or outpatient contacts). The statistical power obtained for numerical outcomes is above 0.98 for Cohen’s D at 0.15, even in the case of a reduction of controls to a 2:1 ratio in the final matching.

### Data collection and outcome measures

#### Outcome measures at 6 months follow-up

The 6 months follow-up will be based on survey data. Both the *headspace* group and the matched control group will be requested via a personal digital e-mail system, to answer an online questionnaire at baseline, which is the first contact with *headspace*, and at the 6 months follow-up. The questionnaires sent to the control group will be administered by Statistics Denmark. There will be a 6 months’ time difference between the *headspace* group and control group’s answers to the same questionnaires because the matching cannot take place until enough *headspace* participants are included in the study. The outcome measures are mental wellbeing, loneliness, life satisfaction and general self-efficacy, measured on the validated psychometric scales: The WHO Five Wellbeing Index (WHO-5) (21), Three-Item Loneliness Scale (18), Cantril’s ladder (16), EuroQol-5 Domain (EQ-5D-5L) (22), and general self-efficacy scale (23). A description of the outcome measures can be found in Appendix 2.

#### Outcome measures for 3 years follow-up

At the 3 years follow-up the following effect of *headspace* will be analysed using register-based data on the following outcomes:

1. Weeks in competitive employment or education in the follow-up period; The Employment Ministry’s longitudinal database (DREAM register data) (24).
2. Hours of work in the follow-up period; The Employment Ministry’s longitudinal database (DREAM register data) (24).
3. Type of education in the follow-up period; Statistics Denmark, highest completed education (UDDA).
4. The proportion receiving cash benefits or early retirement pension at follow-up: the Employment Ministry’s longitudinal database (DREAM register data) (24).
5. Number of admissions in a psychiatric hospital in the follow-up period; National Patient Register (LPR) (25).
6. Number of outpatient visits in psychiatric treatment in the follow-up period; National Patient Register (LPR) (25).
7. The number of social services received according to Danish Social Service Law. This includes personal social support §85, provided social activities §104 and housing for people with mental illness §83 and §85 (Statistic Denmark, Handicap services).
8. Days of school absence in the follow-up period; National Agency for It and Learning.
9. Wellbeing in primary, short-term tertiary and upper secondary school in the follow-up period; Wellbeing surveys in primary, short-term tertiarty and upper secondary school. National Agency for It and Learning.
10. Test scores from the National School tests (Danish and Math) in the follow-up period; National school tests, National Agency for It and Learning.
11. Exam grades in Primary School in the follow-up period; Final grades for short-term tertiary and upper secondary school (UDG), Statistics Denmark.

### Data analysis

#### Sample size

A very conservative estimate of 1,900 participants with complete data at both baseline and follow-up (400 in *headspace* and 1,500 among controls), will provide 90% power to detect standardised mean differences of 0.18 in a two-sided *t*-test. This corresponds to 0.22 points on the WHO-5 Wellbeing Index, 1.16 points on the General Self-Efficacy scale and 0.36 points on Cantril’s Ladder. However, the statistical power might be degraded for analyses relying on multiple imputation because of the expected substantial proportion of missing data. The registry-based outcomes measured at 3 years of follow-up are unaffected by missingness and will have an expected 10,000 controls and 1,500 cases. This will provide statistical power sufficient for detecting small effect sizes, e.g., 83% power for detecting a 12.5 vs. 10% difference on a binary outcome.

Baseline characteristics for the two groups will be reported using means and standard deviations for numeric variables and count (n) with percentages for categorical variables. Mean difference from baseline to follow-up will be presented for both the *headspace* and the control group for all the included outcome measures. For normally distributed continuous outcomes a linear regression will be conducted to test the differences between the two groups. For non-normally distributed outcomes a proportional odds model will be used. The analyses for differences between groups will be adjusted for any baseline differences exceeding a predefined threshold. Moreover, subgroup analyses will be conducted where all analyses will be stratified for age (≥18 years). Missingness will affect the survey measures recorded at baseline and follow-up. Missing data will be handled using multiple imputations, possibly in combination with inverse probability weighting of observations (26). Imputation variables will be included from the complete (or near-complete) and co-occurring registry-based data observations as well as from all survey data collected up to the timepoint of the measure being imputed. A detailed statistical analysis plan (SAP) is also published and available at: https://archive.org/details/osf-registrations-9kzfd-v1.

## Study 2: economic evaluation

The aim of the economic evaluation is to evaluate the cost-effectiveness of *headspace*, compared with a matched control group. The evaluation takes on a health care perspective when considering the costs of *headspace*, including intervention and health care costs. The study uses the same cohort as in the effectiveness evaluation, described above.

### Outcome

Outcome for the cost-effectiveness analysis will be weeks in competitive employment or education during follow-up.

### Costs

The total costs of individuals in contact with *headspace* including intervention costs will be estimated and compared with the costs of individuals in the control group. Non-parametric bootstrapped *t*-test analysis will be used to estimate the statistical significance of the cost difference between *headspace* participants and controls. Mean costs and *p*-values will be reported to show a statistically significant difference between the groups. Intervention costs will be estimated by using information on operating costs from the *headspace* centres.

### Register data

All data will be drawn from Danish national registers. The study will include information on health care services (somatic and psychiatric inpatient and outpatient care, including emergency services and diagnosis related groups (DRG), general practitioners, dentists and other medical specialists, medical prescriptions) and social services. Differences in utilisation of health care will be reported in natural units and costs, while differences in utilisation of social services will be reported in natural units. All elements included in the analyses are described in more detail in Table 2.

**Table 2:** Components included in the economic evaluation.

Costs	Definition	Source
Hospital costs	Inpatient, outpatient, and emergency room contacts in somatic and psychiatric hospitals, valued with Danish national diagnosis-related groups (DRG)-tariffs.	The National Patient Register with DRG and outpatient tariffs (37, 38).
Primary healthcare costs	Contacts to general practitioners, practising specialists and other health care professionals reimbursed (or partly reimbursed) by the Danish National Health Service, e.g., dental care or psychological treatment. Costs are valued with national service tariffs.	The National Health Service Register (39).
Prescription pharmaceuticals	The full price (regardless of subsidies etc) of prescription drugs purchased in Danish pharmacies.	The Pharmaceutical Database (40).
Transfer payments	Cash benefits, early retirement pension, education benefits, other transfers	DREAM database
Costs of labour market interventions	All interventions initiated by the municipal job centres: counselling, mentor support or vocational rehabilitation interventions.	Data will be obtained from the Danish Agency for Labour Market and Recruitment.
Social service costs	Alcohol abuse treatment; drug abuse treatment, day-care; drug abuse treatment; temporary housing; long-term housing; soc. support in housing; public housing; soc.pæd. Support in one’s own home; subsidies for personal and practical help; disability assistance scheme; companion scheme; sheltered employment or activity; and social services	Data will be obtained from Statistics Denmark
Intervention costs	Costs of a counselling session at headspace will be calculated	Data will be obtained from headspace Denmark.
Police, judiciary, and penitentiary	Imprisonment; conviction for violent and moral crimes; conviction for burglary, theft, and vandalism; conviction for traffic law and other special laws	Data will be obtained from the Danish system of criminal statistics
Tax on income	Tax on transfer income, employment	e-income register
Education	Youth education and higher education	Danish Education Registry

### Cost-effectiveness analyses

The time horizon of the evaluation will be 3 years from the first contact to *headspace*. To evaluate cost-effectiveness, the incremental cost-effect ratio (ICER) will be estimated (27). The ICER represents the cost differences between the *headspace* group and the control group divided with differences in outcome measured by labour market contact/educational attainment. Nonparametric bootstrapping will be used to estimate confidence intervals of the mean differences of the groups. Predicted ICERs will be depicted on cost-effectiveness planes to show uncertainty therein. A cost-effectiveness acceptability curve will be generated to assess the probability of *headspace* being cost-effective when the decision-maker is willing to incur additional costs for an extra point increase in outcome up to a given threshold.

## Study 3: process evaluation

The aim of the process evaluation is to investigate the implementation, context, and mechanisms of impact of the key activities in the *headspace* intervention. The focus of the study is the four key activities of *headspace:* (1) individual counselling with *headspace* volunteers, (2) bridging to established social and health services, (3) chat services (counselling and referral to individual counselling) and (4) community engagement where counsellors engage in community activities to obtain visibility regarding *headspace* and possibly also reduce stigma towards mental vulnerability. In line with the MRC guidelines (28), and as recommended by Moore et al. (29), the study will address the three aspects of the *headspace* intervention, implementation, mechanisms of change and context, through a multi-stakeholder perspective (Table 3).

**Table 3:** Overview of research questions and data collection methods in the process evaluation of the headspace intervention.

Aspect of process evaluation	Research question	Data collection methods (what)	Data source (who)	Data collection (how)	Data collection period
Implementation	What is implemented in terms of the key activities counselling, chat counselling, bridging to other services and community engagement?	Forms registering headspace key activities	The forms are filled out by the headspace counsellors after conducting key activities and send to the headspace organisation administration unit	Data on key activities for all headspace centres and displayed for each of the four headspace centres and the chat centre participating in the process evaluation will be sent from the administration unit to the researcher on request.	Data is collected continually in the study period (2023)
	Who are reached?	Forms registration service users	The forms are filled out by the headspace counsellors after counselling and send to the headspace organisation administration unit	Same as above	Data is collected continually in the study period (2023)
	How is delivery achieved?	Participant observation and interviews	Four headspace centres and one national chat centres Managers, counsellors (paid and volunteer), assertive professionals and service users.	Trained research centre attends key activities and other activities in headspace centres (3 days pr. centre) Key informant interviews with managers and assertive professionals Focus group interviews with counsellors at the headspace centres. Service users are recruited for interviews	Data is collected from June 2023 until January 2024
Mechanisms of impact	How do the service users respond to and interact with the intervention?	Participant observation of key activities and interviews	Service users, counsellors	Trained researchers participate in counselling sessions in the 4 headspace centres and in the chat centre when the service user provide consent. Service users are invited to interviews with researchers about their experiences with the headspace intervention	In the study period (June 2023–January 2024)
	How do the key activities create the intended effects on services users’ mental health, loneliness, and self-efficacy?	Interviews	Service users		
	Which unexpected effects/consequences might there be?	Interviews	Service users		
Context	How do contextual factors (legal, organisational, cultural, geographical, and political) affect the implementation and the outcomes of the intervention?	Participant observation, interviews	National manager, cross centre managers, assertive professionals, counsellors	Interviews are conducted in conjunction with participant observations at the headspace centres. If needed key informant interviews will be conducted with additional cross centre managers and service users (until saturation is achieved)	During the study period
	How do services user’s contextual factors affect the implementation and the outcomes of the intervention?	Participant observation, interviews	Counsellors, service users		

### Study participants and data collection methods

The process evaluation will predominantly deploy a qualitative research methodology, as it is suitable for investigating how the headspace intervention is implemented to produce its intended outcomes and how interactions, dynamics, and contextual factors influence implantation (29). This will be done by investigating the stakeholders’ interactions, experiences, and perspectives on the process. The stakeholders in this study are identified as the service users, the implementers, and the management of headspace Denmark. Thus, study participants are young people using headspace counselling, the counsellors (paid counsellors and volunteers) the assertive professionals, and the cross-centre management.

To explore and capture the complexity and nuances of how the intervention is delivered, the data collection will take place in four headspace centres in Denmark, and in the national chat enter and utilise a combination of data collection methods: semi-structured interviews (individual and focus group) and participant observations.

### Implementation

Implementation can be defined as “the process through which interventions are delivered” (29). The delivery of the four key activities (counselling, chat counselling, bridging to other services, community engagement) in *headspace* is described through semi-structured interviews (individual and focus group) and observations of the stakeholders in the intervention: volunteer counsellors, paid counsellors, chat counsellors, assertive professionals, and managers (both cross-centre management and the national *headspace* CEO). Factors that support or impede the implementation of these four activities (e.g., training, knowledge sharing) are investigated. Participant observations will be used to gain a more in-depth understanding of how the activities are practised and how that adds nuances to the described experiences from the interviews (30). The implementation of key activities will also be described through interviews with service users and descriptions of their experiences in the *headspace* intervention. In addition to investigating the intervention delivery processes, the *headspace* intervention’s reach and dose will be described quantitatively with register data from the *headspace* centres.

### Mechanisms of impact

Mechanisms of impact are defined as “the intermediate mechanisms through which intervention activities produce intended (or unintended) effects” for the individual service user (29). This study will describe possible mechanisms by which the key activities might create effects on service users’ loneliness, wellbeing, self-efficacy, and mental health (see Figure 1). The study will emphasise the perspectives of the service users and investigate their (possible) progression after engaging with *headspace.* If feasible, service users will be interviewed twice. The first interview is expected to take place right after the first counselling session. Previous evaluations have shown that service users engage in 2.7 counselling sessions on average (15). Follow-up interviews are expected to be conducted after the last counselling session and on average 4 months after the first session. This relatively short follow-up time (compared to study 1) will ensure that experiences regarding *headspace* are not too far away in memory and thus provide longitudinal information about the life course of each service user and their experienced benefits (or negative experiences from interacting with *headspace* personnel).

### Context

Context is broadly understood as “factors external to the intervention which may influence its implementation, or whether its mechanisms of impact act as intended” (29). Contextual factors that might be relevant for the delivery and function of the *headspace* intervention can both be related to the context of the service user, the political and geographical context, the organisational context, the socio-economic context, legal and cultural context (31). Some variation in the way the *headspace* intervention works in different municipalities with different demographical and organisational settings is expected. To be able to address how these contextual factors might influence the implementation and functioning of the intervention, we will apply a sampling strategy with a high variation (for instance centres located in urban vs. rural areas) between cases (32). By understanding how the intervention possibly works differently or is implemented differently in different municipal contexts can give clues for how context affects implementation and mechanisms of impact (31).

### Data analysis and integration

The quantitative data will be analysed, using descriptive statistics to provide information on the reach and dose of delivery of the *headspace* intervention. The data will be presented for all *headspace* centres in Denmark and the four *headspace* centres and the national chat centre participating in the process evaluation. The qualitative data is recorded digitally and transcribed ad verbatim, using NVivo software. The transcripts will be analysed thematically, using both a deductive approach with the preliminary programme theory as a point of the departure and an inductive approach for allowing unforeseen information and emerging themes to come forward. The findings of the process evaluation will not be reported to the centres continuously or used for quality improvement purposes, as the effectiveness evaluation attempts to evaluate the *headspace* intervention under “natural” circumstances. Thus, this process evaluation can be characterised as summative.

### Ethics statement

The evaluation will be conducted in accordance with the present protocol, the Helsinki Declaration in its latest form, good clinical practise guidelines, and national and EU legislation on data management. The effectiveness evaluation, the cost-effectiveness evaluation and the process evaluation are not expected to pose any known adverse effects. For all studies, informed consent will be utilised in accordance with Danish and European legislation, and all service users who participate in the survey of questionnaires, observations and/or interviews will be secured anonymity (Privacy no. P-2022-602). Since the evaluation is not a biomedical study and because the intervention is conducted at the *headspace* centres, the protocol does not require acceptance from the Ethics Committee in the Capital Region of Denmark (Case number of correspondence with the Ethics Committee: 21061429).

## Discussion

This paper presents the study protocol for three studies: an effectiveness, cost-effectiveness, and process evaluation of the *headspace* intervention in Denmark. Because the *headspace* model has achieved national endorsement and is fully implemented in 30 municipalities throughout Denmark there is an urgent need for scientific knowledge about the effectiveness of the intervention. The evaluation will contribute with new, important knowledge that not only will benefit *headspace* Denmark, but also contributes with new knowledge and documentation targeted to international mental health promotion initiatives, primary and lower secondary schools, educational institutions, and municipal social initiatives.

Overall, the three studies presented in this paper will bring new knowledge about the potential contribution and impact of *headspace Denmark* as a preventive intervention and contribute with new and useful knowledge about the possibly derived effects of counselling sessions in *headspace*. The large number of participants, as well as the establishment of a matched control group and long-term follow-up, makes it possible to provide more reliable and accurate estimates compared with previous studies.

The economic evaluation will contribute new knowledge about the cost-effectiveness of *headspace* Denmark, which can be used for a more nuanced societal prioritisation of resources allocated to vulnerable young people. Finally, the process evaluation will thoroughly describe the key activities of the Danish *headspace* intervention and document how these can be implemented in a local context and how they might create the expected effects on youth mental health and wellbeing. This valuable information can in a future perspective be used to further qualify the *Danish headspace* model.

### Methodological challenges

Despite a comprehensive study design, some methodological challenges exist. The main limitation of this study is the design—that the effectiveness evaluation of the Danish *headspac*e model is not performed as a randomised controlled trial, as it was not practically possible. The second most important limitation is difficulties in identifying a control group. We have tried to solve that by matching on failure to thrive, but we cannot identify the most important variable: help-seeking behaviour. Therefore, there is a risk that participants in the control group might have a better prognosis because they do not feel quite as bad (even though they have the same wellbeing and other match factors). There may also be a risk that participants in the *headspace* group have a better prognosis because they were able to seek and find help. There is also a risk that young people in the control group seek help from *headspace* centres after taking part in the study. Although the members of the control group live outside the catchment areas of the headspace centres, there might be a chance that some individuals contact the centres or the national chat centre during the study period. This introduces a risk of contamination, as both groups will be exposed to the intervention investigated. Young people in the control group who need help can use available social and health services, and mental health support offered in their community, but no signposting to specific organisations/services is provided in the material sent to the participants.

A potential pitfall in the effectiveness evaluation pertains to selection bias and the dynamics of outcomes before participation in the study (33). Young people’s mental health and wellbeing may be volatile with emotional ups and downs rather than persistent with the same levels of wellbeing over time, for instance shown in a study by Tegner Anker et al. (34). Youth would primarily seek help at “mental downturns,” that is at times with high level of emotional distress, there will be a regression to the mean, that might be difficult to identify in the control, thus posing a risk that natural improvements will be interpreted as effectiveness of the *headspace* intervention. While analysing the data, differences between the groups on all baseline outcomes will be tested for statistical significance. The analyses for differences between groups will be adjusted for any baseline differences with a *p*-value below 0.05.

Participants in the *headspace* group and the control group are matched on individual-level characteristics only. At the beginning of the data collection period, there were headspace centres in 22 out of the 100 municipalities of Denmark, meaning that it was not hard to identify controls. There were headspace centres is the four largest cities of Denmark (Copenhagen, Aarhus, Odense, and Aalborg) but not in some of the other larger cities such as Silkeborg or Kolding. There could be unobserved municipality-level factors potentially affecting the treatment effect estimates, e.g., related to urban density or distance to urban centres. We have, however, seen no substantial problems for the matching algorithm to identify comparable controls on the observed factors. We would also, from a pragmatic point of view, expect important municipality-level differences to be captured by the range of individual-level matching factors used (e.g., social status).

A limitation in the effectiveness evaluation may be that not all the register-based data will be completely updated at the time of matching. However, historical data such as national school tests, and wellbeing at primary school is expected to be prognostic and relevant to use in the matching. Moreover, many but not all the registers are updated in real-time such as the National Patient Register, which is available from The Danish Health Data Authority after a few days. Moreover, the matching procedure will be repeated before conducting the 3 years effect analyses, where all data will be updated at the time of matching. A limitation in using surveys as data collection methods is that respondents constitute a sample of the population rather than the whole population of *headspace* service users. As *headspace* collects basic information on service users, a supplementary analysis will be performed, comparing *headspace* users accepting registration on unique personal ID with *headspace* users not accepting to give their personal ID and participate in this evaluation. The comparison includes age, gender and additional information on social and health-related problems faced by participants. The process evaluation must consider, the flexibility of the *headspace* intervention. Key principles in the *headspace* model necessitate a person-centred approach where the consultation is guided by what is on the mind of the young person seeking support. Also, the counsellors are encouraged to take ownership in the local *headspace* centres and initiatives to develop local services. Although the sampling strategy is based on maximal variations with respect to factors such as urban/rural locations, new/old centres and high/low community engagement, there might be other important factors not considered in the selection of centres that affect how the *headspace* intervention is implemented and works, thus comprising the external validity of the findings.

Also, the evaluation’s limitation pertains to potential bias in the volunteer-based counselling model offered in the *headspace* centres, which may affect issues related to the outcomes and implementation. The young people seeking counselling in *headspace* may be characterised by distinct features (e.g., related to motivations, and skills) compared to the whole population of youth with mental health problems. Moreover, the young people who participate in the studies might be different from the group of service users in *headspace.* They may already have positive attitudes and expectations towards counselling. This potential selection bias might lead to an overestimation of the effectiveness of the counselling offered in *headspace* (35). Another limitation of the volunteer-based counselling model is that the counsellors posit varying levels of skills and competencies, which may lead to inconsistent quality of counselling. Although training is provided to the counsellors before and during their involvement in *headspace,* there is a risk that the effectiveness of the counselling model depends on the counsellors` experiences rather than the model itself. To ensure that the headspace intervention is implemented as intended and strengthen the quality of the counselling services provided in headspace centres, a fidelity scale will be developed based on the findings from the process evaluation (36).
