# Supplementary material for: Study protocol: an effectiveness, cost-effectiveness, and process evaluation of headspace Denmark
Source: Front Public Health. 2025 Apr 7;13:1491756. doi: 10.3389/fpubh.2025.1491756 (PMC12009928; doi:10.3389/fpubh.2025.1491756)
Supplement: Supplementary file 1 [file Supplementary_file_1.docx]

Appendix 1: Timeline of the *headspace* Evaluation

| **Study** | **2022** | | **2023** | | | | **2024** | | | | **2025** | | | | **2026** | | | |
| --- | --- | --- | --- | --- | --- | --- | --- | --- | --- | --- | --- | --- | --- | --- | --- | --- | --- | --- |
|  | Q3 | Q4 | Q1 | Q2 | Q3 | Q4 | Q1 | Q2 | Q3 | Q4 | Q1 | Q2 | Q3 | Q4 | Q1 | Q2 | Q3 | Q4 |
| **Study 1**  Effectiveness evaluation | Inclusion  Matching  Data collection of 6 moths outcomes | | | | | | | | | Analysis  and report  of 6 months'  outcomes | |  |  |  |  | | Analysis  and report  of 3 years  outcomes | |
| **Study 2**  Economic evaluation | Inclusion  Matching (same cohort as study 1) | | | | | | | | |  |  | | | |  | | Analysis  and report  of 3 years  outcomes | |
| **Study 3**  Process evaluation | Data collection in 4 *headspace* centers | | | | | | | Analysis | | | | Report findings on implementation and mechanism of impact | |  | | | | |
