# Supplementary material for: Study protocol: an effectiveness, cost-effectiveness, and process evaluation of headspace Denmark
Source: Front Public Health. 2025 Apr 7;13:1491756. doi: 10.3389/fpubh.2025.1491756 (PMC12009928; doi:10.3389/fpubh.2025.1491756)
Supplement: Supplementary file 2 [file Supplementary_file_2.docx]

Appendix 2: List of outcome measures

**WHO-5**

The 5-item World Health Organization Well-Being Index (WHO-5) is a short and generic global rating scale measuring subjective well-being. The scale consists of 5 items: (1) *‘I have felt cheerful and in good spirits’, (2) ‘I have felt calm and relaxed’, (3) ‘I have felt active and vigorous’, (4) ‘I woke up feeling fresh and rested’ and (5) ‘My daily life has been filled with things that interest me’.* Respondents are asked to rate how well each of the 5 statements applies when considering the last 14 days. Each of the 5 items is scored from 5 (all the time) to 0 (none of the time). The raw score ranges from 0 (absence of well-being) to 25 (maximal well-being). It is recommended to multiply the raw score by 4, as scales measuring health-related quality of life are conventionally translated to a percentage scale from 0 (absent) to 100 (maximal).^1^

**Three-Items Loneliness Scale**

The Three-Items Loneliness Scale (TILS) is a three-item self-reported measure. The 3 items are *(1) how often do you feel that you lack companionship? (2) How often do you feel left out? (3) How often do you feel isolated from others?* The respondents are asked to rate each item on a 3-point Likert scale coded 1 ‘hardly ever’, 2 `some of the time` or 3 ‘often’. Responses are summed up, with higher scores indicating greater loneliness (range from 3 to 9).^2^

**The Cantril`s ladder**

The Cantril ladder measures life satisfaction by asking the respondent (1) to imagine their life in the best possible light and to describe their hopes and wishes for the future, (2) to imagine their life in the worst possible light and describe their fears and worries associated with this. Then, the respondent is presented to a ladder numbered from 0 at the bottom to 10 at the top, with the following text: ‘*Here is a picture of a ladder. Suppose the top of the ladder represents the best possible life for you and the bottom of the ladder the worst possible life. Where on the ladder do you feel you stand at the present time?* (Ladder present) Respondents are also asked to rate where they will be 5 years from now. (Ladder future) ^3^

**General self-efficacy scale**

General self-efficacy scale (GSE) is a self-reported measure, consisting of 10 items: *(1) I can always manage to solve difficult problems if I try hard enough, (2) If someone opposes me, I can find the means and ways to get what I want, (3) It is easy for me to stick to my aims and accomplish my goals (4) I am confident that I could deal efficiently with unexpected events (5) Thanks to my resourcefulness, I know how to handle unforeseen situations (6) I can solve most problems if I invest the necessary effort, (7) I can remain calm when facing difficulties because I can rely on my coping abilities (8) When I am confronted with a problem, I can usually find several solutions (9) If I am in trouble, I can usually think of a solution (10) I can usually handle whatever comes my way.* The respondents are asked to rate each item on a 4-point Likert scale, ranging from 1 (not true at all) to 4 (exactly true). The total score is calculated by finding the sum of all items, and ranges between 10 and 40, with a higher score indicating more self-efficacy.^4^

**The** **5-level EQ-5D version**

The 5-level EQ-5D version (EQ-5D-5L) measures health related quality of life. The respondent is asked to tick of their health status withnin five dimmensions**:** mobility, self-care, usual activities, pain/discomfort and anxiety/depression. Each dimension has 5 levels: no problems, slight problems, moderate problems, severe problems and extreme problems. Higher scores indidicate worse possible state. The scores can be aggregated into a utility score from a 0-1 scale. In addtion, respondents rate their general health related quality of life on a visual analogue scale (EQ VAS) from 0 (“worst imaginable state of health”) to 100 (“best imaginable state of health”).^5^

References:

1. Topp, C. W., Østergaard, S. D., Søndergaard, S. & Bech, P. The WHO-5 Well-Being Index: A Systematic Review of the Literature. *Psychother. Psychosom.* **84**, 167–176 (2015).

2. Hughes, M. E., Waite, L. J., Hawkley, L. C. & Cacioppo, J. T. A Short Scale for Measuring Loneliness in Large Surveys. *Res. Aging* **26**, 655–672 (2004).

3. Levin, K. A. & Currie, C. Reliability and Validity of an Adapted Version of the Cantril Ladder for Use with Adolescent Samples. *Soc. Indic. Res.* **119**, 1047–1063 (2014).

4. Scholz, U., Doña, B. G., Sud, S. & Schwarzer, R. Is general self-efficacy a universal construct? Psychometric findings from 25 countries. *Eur. J. Psychol. Assess.* **18**, 242–251 (2002).

5. Jensen, M. B. *et al.* Danish population health measured by the EQ-5D-5L. *Scand. J. Public Health* **51**, 241–249 (2023).
